# Patent Medicine

**Published:** 1883-07

**Authors:** A. L. Craig

**Affiliations:** Galesburg, Ill.


### ARTICLE VII.

PATENT MEDICINE. By A. L. CRAIG, M.D., Galesburg, Ill. Read before the Military Tract Med. Association, May 8, 1883.

It must be that to-day we are witnessing the ushering in of the patent medicine era.

For years this branch of business has been gradually and rapidly assuming the immense proportions which it now presents.

It has been so successfully managed that it has become a recognized trade, and millions of money have been invested in this kind of enterprise. That, as a business, it has been immensely successful is affirmed by the appearances of wealth of its enterprising managers, as exhibited in their fine palatial mansions and beautiful grounds, their large, substantial laboratories and manufactories, and in the extravagant manner in which they advertise their nostrums.

It is a traffic which annually wrings millions of money from the suffering ignorant and credulous, and offers but little in return but disappointment and chagrin.

It is a most fortunate thing for the patent medicine business that the world is one of disease and suffering, and the proprietors of these nostrums are fully aware of the fact that one who is sick or suffering will turn where there is the most encouragement offered. No means are spared to place their nostrums on the market; no means that money can provide is left untried. The best of literary talent is enlisted and paid extravagently to write advertisements, sensational circulars, pamphlets, and even certificates.

Men are continually racking their brains for some new and novel way for the more showy and attractive manner in which to catch the public eye.

Their fallacious advice is to be found intermingled with more or less useful and instructive reading in the yearly distribution of almanacs, of which millions are annually scattered broadcast, in all languages of the civilized world. Rocks, buildings and fences are covered with glittering advertisements, and we are told that even the flag of our Union has been prostituted for this purpose. Our secular, religious, and even the medical press, are subsidized in the interest of this trade, and, finally, it must be admitted that certificates, purporting to come from medical men, are published, claiming to have used and prescribed certain of these remedies with gratifying results.

Is it any wonder that, after all the inducements held out by these advertisements in the shape of circulars and almanacs—especially the latter—telling, as the people say, "just how I feel," followed by the certificates of medical men—in many cases trumped up, no doubt, and, too, by the prescribing of them by members of the profession—is it any wonder, I ask, that the masses invest their money in such goods?

I think not, as more is guaranteed by them than any honest practitioner could promise.

As I said a moment ago, certainly we are about to witness a new era in the patent medicine business. A new impetus has been given the trade by the recent abolishment of the revenue tax.

A movement was instituted in the winter of 1881 and 1882, by Vogeler & Co., prominent proprietary medicine men of Baltimore, for the purpose of securing the repeal of the revenue tax on patent medicine, perfumes, etc., setting forth the claim that the tax was onerous, and that its repeal was fully justifiable; that it was a war levy, and the exigency having passed, the tax should no longer remain.

It was further set forth that these goods were not luxuries, but necessities, inasmuch as as they represented by far the greatest amount of medicine used in this country.

Also that, as the revenue existed, it was really a double taxation; first on the raw material, and then in the manufactured state, amounting to 8 or 10 per cent.

It was hoped that Congress would pay no attention to this memorial. But with the press subsidized to the extent it is, it was hoping for too much, and with the combined influence of the press and the lobby, the repeal was effected, and the new law goes into effect either the first of July or October next.

The proprietary medicine tax of late years has amounted to nearly two millions of dollars annually, and the repeal of this tax will accrue to the benefit of the proprietor only, as all jobbing houses have been notified that there will be no change in the price of their medicines.

I look upon the new law as an infamous act, and as the outgrowth of corrupt legislation. This business is beginning to assume immense proportions. It is making tremendous strides with each succeeding year, and as it looms up before us, its colossal appearance is enough to make legitimate pharmacy and medicine quail and look about for a remedy against this certain enemy, and for a new lease of life.

As matters exist at present, and in the direction they are certainly tending, it is only a question of time when pharmacy will be pushed to the wall, and the business of the manufacture and prescribing of medicine will become a mere nothing compared with what it ought to be and shonld be; when drug stores will be scarcely anything else than a depot for patent medicines, and any ignoramus a competent so-called druggist.

I do not doubt that some of our patent medicines are possessed of some virtue, and if properly recommended by their proprietors for certain restricted cases, would offer the purchaser a fair assurance that he was getting what he really wanted and needed.

But the wholesale recommendation of each kind of medicine for nearly every ill embraced in the catalogue of diseases, constitutes a fraud of the first magnitude.

Now, in view of what has been said, the question arises, what shall we do to be saved? What kind of steps shall we take to stop this traffic, and curtail it of its present dimensions, and abort the influence it has over the American people?

I say American, because the manufacture and taking of patent medicines is peculiarly a custom of this country.

It cannot be denied that this abuse has crept very largely into American life, and no doubt, like some other of our social evils, is the result of our large and rapid development, and finally as a result of the enterprising and energetic way in which these remedies have been placed and maintained before the public; notices of all their virtues being in all our papers, magazines and journals; circulars and certificates carried by mail and messenger to our hearthstones, and posters staring us in the face wherever we go, in town, country, forest or plain—all heralding to the suffering a certain and speedy cure for all pain and disease.

How are we going to head off this kind of thing, and abort its influence? I confess I hardly know. We have an immense moneyed power to battle with; an interest that acknowledged, in their memorial to Congress, when praying for the repeal of the tax, that in the last twenty years they had paid the press $100,000,000; and of course the bulk of this amount has been spent in the last few years.

Vogeler & Co., the St. Jacob's oil men, spent, themselves, last year, over $400,000 in advertising their remedy.

Druggists cannot fight them by refusing to handle their goods, because they are manufactured for sale, and the proprietors have the money to push them, and the refusal to handle them would only be to drive the trade to some other class of business men, and would not result in the decrease of the sale of such goods.

I do not see that the medical fraternity can wield very much influence in this matter, as every intelligent citizen knows that a doctor, with but few exceptions, will never advise any one to use a patent medicine, or any kind of secret remedy—and more than that, people will often consult a physician concerning some ailment, use his prescription a few days, and then commence with a patent remedy, buy it in half-dozen lots, and take it for weeks uncomplainingly.

In speaking against the use of patent medicines, I do not want to be understood as waging war at the same time against trade-mark preparations, although, in the main, I think they should be passed by in prescribing.

I do think, however, that the trade-mark system is an unfortunate thing for us all, and hope it will not be long before it will be a thing of the past. We have quite a number of trade-mark preparations which have been and are being prescribed daily with gratifying results. The formulæ of some of these remedies have been made known, and the rest should follow suit. The trade-mark is copyrighted, and is thus protected, and as a matter of fact, no harm can befall the proprietor if he make known the exact formula of the remedy and its mode of preparation. It should be done for the benefit of science, and, too, its formula being known will, in most cases, add to its popularity and increase its use.

The patent law, instituted as it was for the stimulation of inventive genius, protected them for a limited length of time, thus affording them opportunity of reaping somewnat of reward for their labors, is undoubtedly a beneficial law. This kind of law is a good thing, because it lays before the people the result of labor which will be protected, and compels one to improve on the article before them if they desire to cope successfully in the sale of their goods.

Not so with the law as it pertains to patent medicines. The secret is in the ownership of a name, and the composition and art of manufacture of the medicine is entirely unknown.

The patent law, as properly applied to pharmacy, must necessarily be productive of benefit, inasmuch as the limited control of processes and machinery for the manufacture, provided knowledge thereof for the benefit of pharmacy and science was fully published, would stimulate the invention of implements to attain the same end, and tend to discourage the manufacture of secret remedies.

Medical men should refrain from the use of any of the patent remedies, or from recommending them, and should also be much more careful and judicious in the use of trade-mark preparations, and in this way help to discourage the secrecy in the manufacture of medicine. But, in order to break down the demand and finally call a halt in the sale and use of patent medicine, it would appear to me that we must go back to the young and rising generation—to our schools—and see if we cannot find a remedy. It is useless to talk to the adult concerning the evil. You can 't teach an old dog new tricks; we must, therefore, turn our attention to the young ones.

I think the great secret of the success of the patent medicine business is to be found in the fact that, as a people, we are profoundly ignorant concerning our physical needs; how we are constituted; what, in brief, are the functions of our principal vital organs, and how we should take care of ourselves.

We know too little of hygeine, physiology and anatomy. Now, I think that in our common schools is the place to lay this kind of knowledge before the people. Give the young an insight of the nature of their physical being and what that being needs for its best preservation and existence.

Let them have this kind of knowledge as they advance to the life of an adult. Let us have a little more of this kind of education, and less of the higher mathematics, of the modern and ancient languages, of music and the fine accomplishments—all very well in their place, and highly necessary for the full compliment of our high grade of social life, but they certainly should not crowd out and supplant the more practical and vital knowledge. I don 't see any other way to stay the progress of this evil than by teaching the people what they need. And when they reach that period, when they come to have some knowledge concerning their needs and the means best adapted to their wants, they will refuse to use a secret nostrum. It will be repugnant to them, as it will not appeal to their sense of knowledge and prudence. It will not be in consonance with their teaching, and rather than buy something of which they have no knowledge whatever, they will buy some non-secret remedy or consult an intelligent physician.

We must look for a higher grade of education among the people, and also for more proficiency in the profession, before we can hope to see the final dissolution of the patent medicine traffic. It will not come before we have legislation doing away with the patenting of formulæ, and the system of trade-marks has been frowned down. In my opinion, we can only hope for this transition when the people have learned more fully what they need and want than they know to-day.

